# Appropriateness of Prophylactic Surgery for Sigmoid Volvulus in View of Recurrence and Nutritional Status

**DOI:** 10.7759/cureus.98429

**Published:** 2025-12-04

**Authors:** Yoshifumi Watanabe, Mitsuo Tokuhara, Hiroko Nakahira, Yasumasa Sumitomo

**Affiliations:** 1 Department of Gastroenterology, Hoshigaoka Medical Center, Hirakata, JPN

**Keywords:** complication, endoscopy, guideline, nutrition, outcome, sigmoid volvulus, surgery

## Abstract

Background

Sigmoid volvulus causes colonic obstruction and ischemia. Urgent surgery is required for colonic necrosis or perforation, whereas prophylactic surgery is recommended after endoscopic treatment in nonemergency cases. However, many patients with sigmoid volvulus only undergo endoscopic treatment, highlighting a gap between guideline recommendations and clinical practice. We aimed to elucidate the actual management and outcomes of sigmoid volvulus in hospitalized patients to reconsider the disadvantages of delaying prophylactic surgery.

Methods

We retrospectively recruited patients with sigmoid volvulus who were treated at the Hoshigaoka Medical Center between January 2013 and September 2024. Participant demographics and outcomes were analyzed, and the probability of remaining recurrence-free was estimated using Kaplan-Meier analysis. Wilcoxon signed-rank tests were used to compare paired samples.

Results

We enrolled 28 participants with sigmoid volvulus. Of these, 15 (53%) had a medical history of cerebral stroke, and 12 (42%) were institutionalized. No participants who underwent surgery experienced recurrence, whereas 10 (62%) of 16 participants who received only endoscopic treatment did. Among patients with recurrence, one died as a result. Body weight, serum albumin level, geriatric nutritional risk index, and prognostic nutritional index were significantly lower at the time of recurrence than at the first hospitalization. No deaths occurred due to postoperative complications.

Conclusions

Sigmoid volvulus frequently recurs after endoscopic treatment alone, posing a life-threatening risk with each recurrence. Most patients with sigmoid volvulus are frail, and their nutritional status often deteriorates before recurrence occurs. Prophylactic surgery should therefore be planned for sigmoid volvulus soon after endoscopic treatment for the first time.

## Introduction

Colonic volvulus is the most common cause of benign mechanical obstruction, accounting for 1.2-7.0% of all cases of colonic obstruction [1,2]. Most cases of colonic volvulus (60-75%) occur in the sigmoid colon, followed by the cecum (25-40%), with cases in the transverse colon and splenic flexure being rare (1-4% and 1% incidence, respectively) [3]. Sigmoid volvulus is caused by rotation of the sigmoid loop by >180° around its meso-axis [4]. This twisting forms a volvulus that traps intestinal contents and becomes enlarged, causing not only colonic obstruction but also necrosis and perforation due to ischemia of the twisted colon. The mortality rate in emergency surgery for sigmoid volvulus has been reported to be up to 16%; thus, the condition requires quick and appropriate treatment [5].

Management of sigmoid volvulus consists of three stages: assessment of colonic viability due to closed-loop obstruction, relief of the obstruction, and prevention of recurrence. Contrast-enhanced CT is the most important examination for diagnosis and for assessment of colonic necrosis and perforation. Studies have shown CT to confirm diagnoses of sigmoid volvulus with nearly 100% sensitivity and >90% specificity [6]. Endoscopy is helpful for confirming colonic mucosal color in the closed-loop obstruction for assessment of viability, and endoscopic decompression and detorsion is a common approach for patients with sigmoid volvulus who are stable without colonic necrosis or perforation. In such cases, the success rate of endoscopic treatment is up to 95% [7]; however, this treatment is only a temporary solution, and the recurrence rate after endoscopic treatment is estimated to be 50-85% [6-8]. Mortality after nonoperative management following endoscopy ranges from 9% to 36%; therefore, elective prophylactic surgery is recommended after endoscopic treatment [3,6,7,9]. Despite this recommendation, only 16.4-33.7% of patients undergo prophylactic surgery after successful nonoperative detorsion, indicating considerable divergence between the recommendation and real-world treatment practices in the context of sigmoid volvulus [5,8,10]. The present study aimed to examine the management and outcomes of patients hospitalized for sigmoid volvulus, including reasons for not performing prophylactic surgery, recurrence rate, and nutritional status, to reassess the disadvantages of delaying prophylactic surgery.

## Materials and methods

Participant recruitment and study population

We recruited all consecutive patients with sigmoid volvulus who were treated at Hoshigaoka Medical Center between January 2013 and September 2024 for this retrospective study. Sigmoid volvulus was diagnosed based on CT findings. The characteristic CT findings of a dilated sigmoid colon, a bird’s beak sign at the point of torsion, and a mesenteric whirl sign were used for diagnosis [4].

Treatment procedures

A flexible endoscope was used for the treatment of sigmoid volvulus. Endoscopists inserted the endoscope into the twisted colonic segment and suctioned trapped gas in the dilated colonic segment for decompression. If possible, the endoscope was advanced to the splenic flexure to create detorsion, confirmed by straightening of the sigmoid colon. A colorectal tube was placed after endoscopic decompression and detorsion according to each endoscopist’s preference.

**Figure 1:**
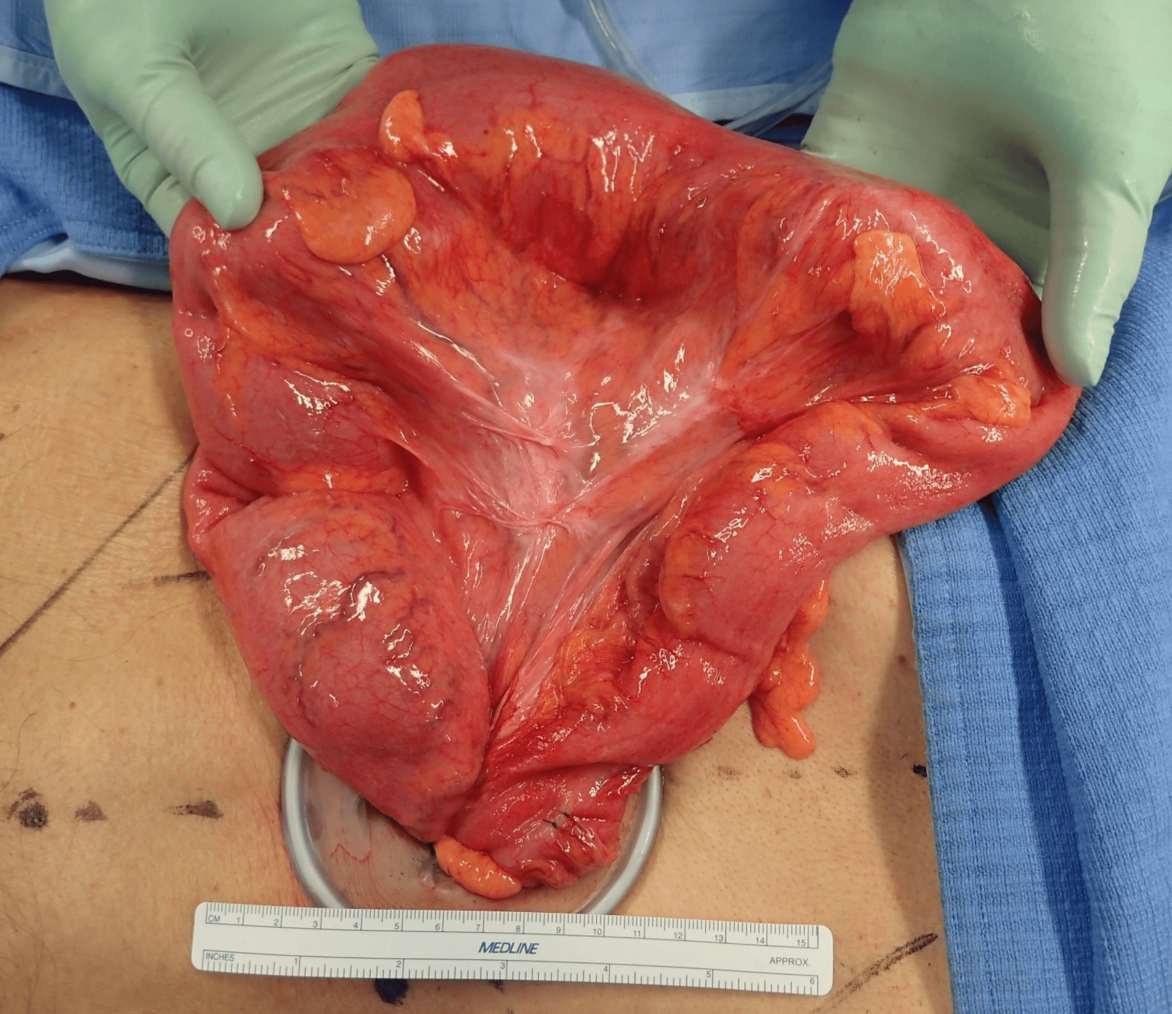
Operative findings Representative clinical photograph showing how the redundant sigmoid colon can be brought out of the body through an incision.

Regarding surgical procedures, two types of surgery were performed: Hartmann’s operation (without intestinal anastomosis) or sigmoid colectomy (with primary intestinal anastomosis). Any redundant sigmoid colon was removed as completely as possible through an incision and resected (Figure 1).

The decision of whether to perform anastomosis was made by the attending surgeons based on the patient’s condition and intraoperative findings.

Data collection and analysis

Data collection and subsequent analyses were performed in November 2024. We contacted patients who did not attend follow-up regularly by telephone to inquire about their clinical course. Clinical data, including age, gender, weight, BMI, Eastern Cooperative Oncology Group performance status, history of past illness, use of medication for constipation, therapeutic procedures, complications, mortality, and recurrence, were collected.

Regarding medical treatments for constipation, we collected data on the history of use of osmotic and stimulant laxatives, secretagogues, ileal bile acid transporter inhibitors, 5-hydroxytryptamine 4 agonists, and Kampo medicine. Probiotics and pantethine were excluded from analysis. We retrieved medical record data on stool frequency and the number of medications for constipation before and after surgery.

To evaluate nutritional status, we calculated the geriatric nutritional risk and prognostic nutritional indices (GNRI and PNI, respectively) [11,12].

The GNRI was calculated using the formula:

$\text{GNRI} = (14.89 \times \text{serum albumin (g/dL)}) + \left(41.7 \times \frac{\text{weight (kg)}}{\text{ideal weight (kg)}}\right)$

The PNI was calculated using the formula:

$\text{PNI} = (10 \times \text{serum albumin (g/dL)}) + (0.005 \times \text{total lymphocyte count (/mm}^3))$

Postoperative complications were classified according to the Clavien-Dindo classification [13].

Statistical analysis

The probability of remaining recurrence-free following surgery or conservative management was evaluated using Kaplan-Meier curves and the log-rank test. Wilcoxon signed-rank tests were used to compare paired samples. Two-sided P values < 0.05 were considered statistically significant. All statistical analyses were performed using JMP Pro 17 statistical software (SAS Institute, Cary, NC, USA).

## Results

A total of 28 patients with sigmoid volvulus were included in this study, with a median follow-up duration of 24 months. The characteristics of the study population are shown in Table 1.

**Table 1:** Characteristics of the patients (n = 28)

Characteristic	Value
Median age (range, years)	78.5 (47-95)
Sex (male/female)	20/8
BMI (range)	20.1 (13.8-31.1)
Performance status score (range)	3 (0-4)
Institutionalized	12 (42%)
Median serum albumin (range, g/dL)	3.7 (1.9-4.6)
Cerebral stroke	15 (53%)
Parkinson’s disease	3 (10%)
Vertebral or lower limb fracture	6 (21%)
Diabetes	5 (17%)
Psychiatric disorder	1 (3%)
Past history of sigmoid volvulus	6 (21%)
Medication for constipation	24 (85%)

The median age at first hospitalization was 78.5 years (range: 47-95 years), and 20 (71%) of the patients were male. The median BMI was 20.1 (range: 13.8-31.1). Most patients had low activity, with a median performance status score of 3, and 12 (42%) were institutionalized. More than half of the patients had a history of cerebral stroke, three (10%) had Parkinson’s disease, and six (21%) had vertebral or lower limb fractures. A past history of sigmoid volvulus prior to the first admission to our hospital was present in six (21%) of the patients, and 24 (85%) were receiving medication for constipation.

**Figure 2:**
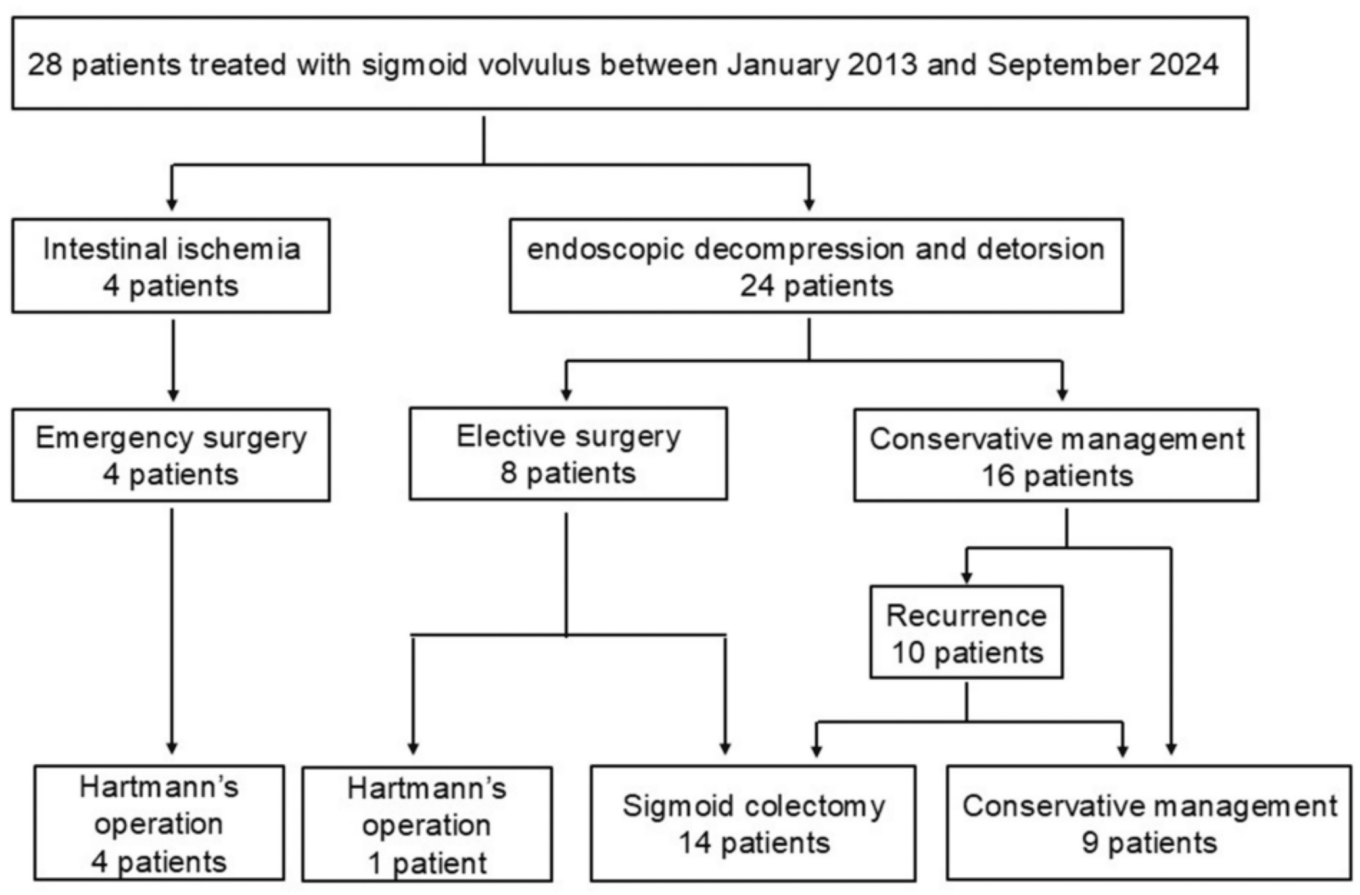
Flow diagram illustrating the protocol for treatment of sigmoid volvulus

A summary of the therapeutic procedures is shown in Figure 2.

Four patients (14%) underwent emergency surgery due to colonic necrosis (two patients) or perforation (two patients). Endoscopic decompression and detorsion were performed in 24 patients (86%), and eight patients underwent elective prophylactic surgery. The remaining 16 patients were followed up without surgery. Reasons for not performing prophylactic surgery at the first hospitalization are summarized in Table 2, with the primary reason being the absence of a surgical proposal, favoring conservative management until recurrence.

**Table 2:** Reasons for not performing elective prophylactic surgery at first hospitalization (n = 16)

Reason	Number of patients
Lack of a surgical proposal (favoring conservative management until recurrence)	5
Priority of treatment for other diseases	5
Patient’s refusal of surgery	4
Old age	1
Unidentifiable	1

Recurrence occurred in 10 of 16 participants who did not undergo surgery at first hospitalization, and seven of these subsequently underwent prophylactic surgery after endoscopic retreatment (four of five patients without a surgical proposal at first hospitalization had recurrence and subsequently underwent surgery, while three of four patients who initially refused prophylactic surgery had recurrence, and one of these subsequently underwent surgery). By the end of follow-up, 19 (67%) of all participants had undergone surgery.

Among the 15 patients who underwent elective prophylactic surgery, the median time to surgery was nine days (range: 3-82) after endoscopic treatment. Regarding surgical procedures, 11 (73%) of 15 participants underwent laparoscopic surgery in elective cases, whereas open surgery was performed in all emergency cases. Almost all participants who underwent elective surgery (14 of 15) underwent sigmoid colectomy, while Hartmann’s operation was performed for all emergency cases (Figure 2).

**Figure 3:**
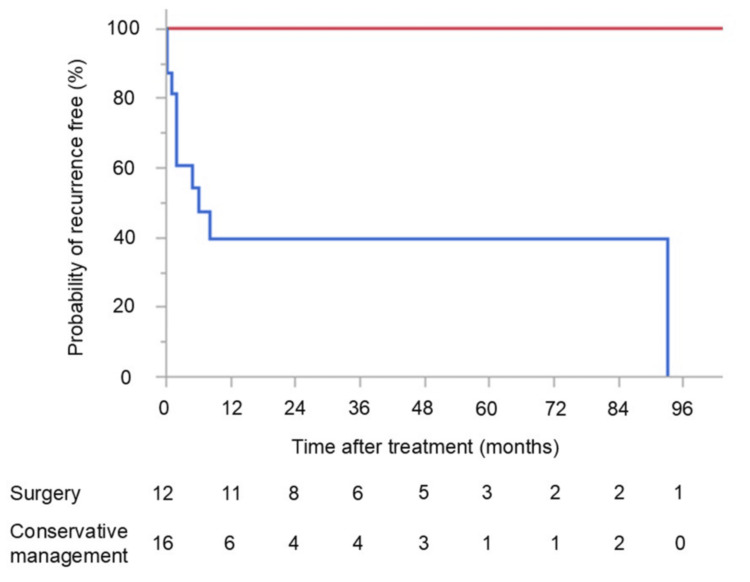
Probability of remaining recurrence-free after surgical or conservative management No participants who underwent surgery experienced recurrence; however, those who did not undergo surgery often experienced recurrence within 12 months of endoscopic treatment. The recurrence rate was significantly higher among participants who did not undergo surgery compared with those who did (P < 0.05). Red line: surgery; blue line: conservative management

Figure 3 shows the probability of remaining recurrence-free among participants who underwent surgery and those who did not at the first hospitalization.

No participants who underwent surgery experienced recurrence, but those who did not undergo surgery often experienced recurrence within 12 months of endoscopic treatment. The recurrence rate was significantly higher among participants who did not undergo surgery compared with those who did (P < 0.05). Eight participants, three who did not undergo surgery and five who did, died during the follow-up period. Patients who underwent surgery had no disease-specific deaths, whereas a disease-specific death occurred in one patient (6%) of the 16 who did not undergo surgery at first hospitalization. This patient presented to our hospital in shock and subsequently died from recurrent sigmoid volvulus.

**Figure 4:**
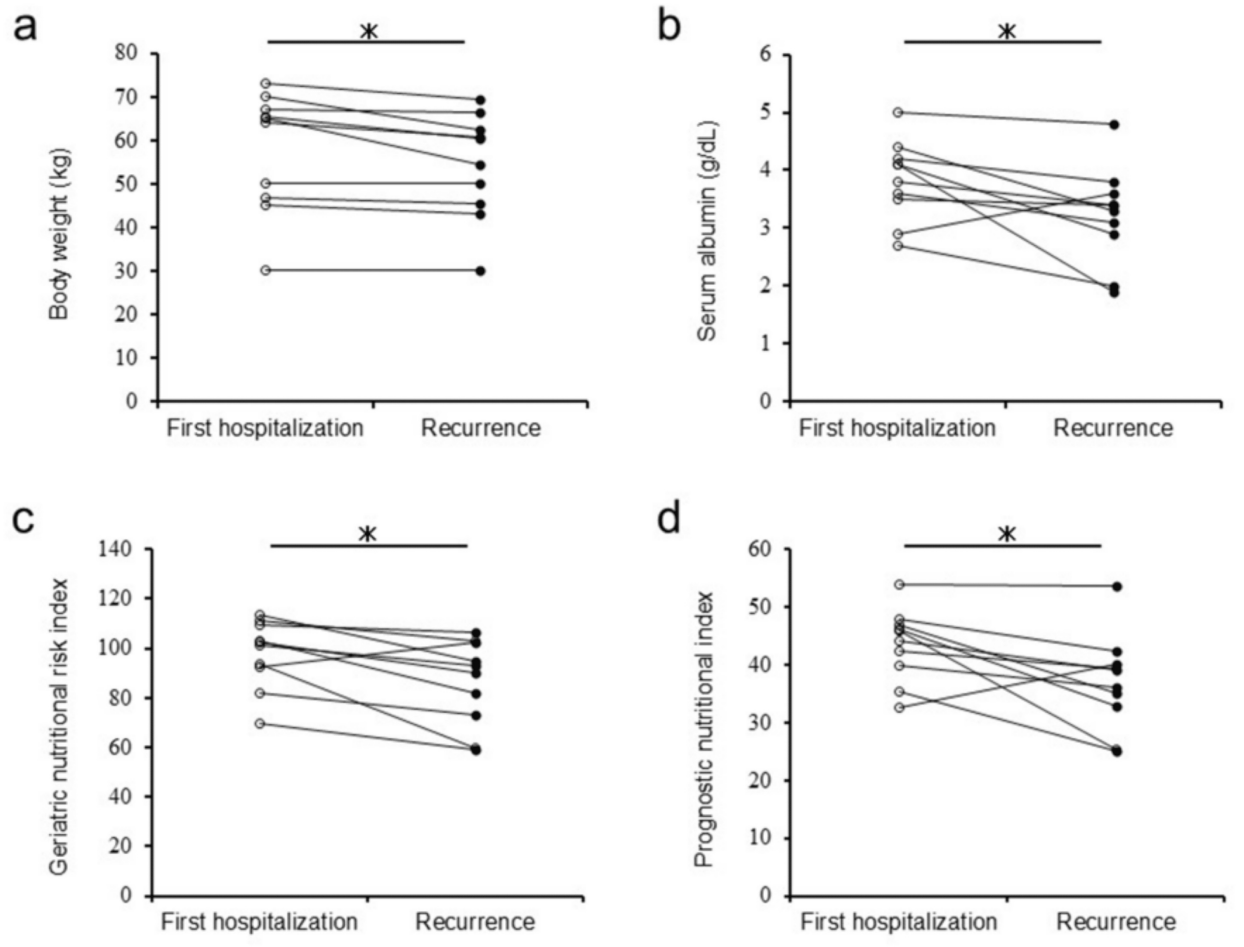
Graphs comparing nutritional status at the time of first hospitalization and at recurrence (a) Body weight (kg). (b) Serum albumin (g/dL). (c) GNRI. (d) PNI. GNRI, geriatric nutritional risk index; PNI, prognostic nutritional index ^*^ P < 0.05

Regarding patients’ nutritional status, body weight, serum albumin level, GNRI, and PNI were significantly lower at recurrence compared with values at first hospitalization (P < 0.05) (Figure 4).

The median serum albumin levels were 3.95 g/dL at first hospitalization and 3.35 g/dL at recurrence. For GNRI and PNI, the median values were 101 and 45, respectively, at first hospitalization, compared with 91 and 37 at recurrence.

Details of postoperative complications are summarized in Table 3.

**Table 3:** Postoperative adverse events

Complication	Emergency (n = 4)	Elective (n = 15)
Surgical site infection, n (%)	1 (25%)	0 (0%)
Ileus, n (%)	2 (50%)	3 (20%)
Pneumonia, n (%)	2 (50%)	2 (13%)
Urinary tract infection, n (%)	1 (25%)	1 (6%)
Hemorrhage, n (%)	0 (0%)	1 (6%)
Thrombosis, n (%)	1 (25%)	0 (0%)
Dysphagia, n (%)	1 (25%)	0 (0%)
Clavien-Dindo classification		
Grade I-II, n (%)	4 (100%)	7 (46%)
Grade III-IV, n (%)	2 (50%)	0 (0%)
Grade V, n (%)	0 (0%)	0 (0%)

Overall, the rate of complications was higher among patients who underwent emergency surgery, with ileus, pneumonia, and urinary tract infection being the most common adverse events in both emergency and elective surgeries. No anastomotic leakage occurred. All postoperative complications in elective surgery were grade I or II according to the Clavien-Dindo classification. No patients died due to postoperative complications following either emergency or elective surgery.

**Figure 5:**
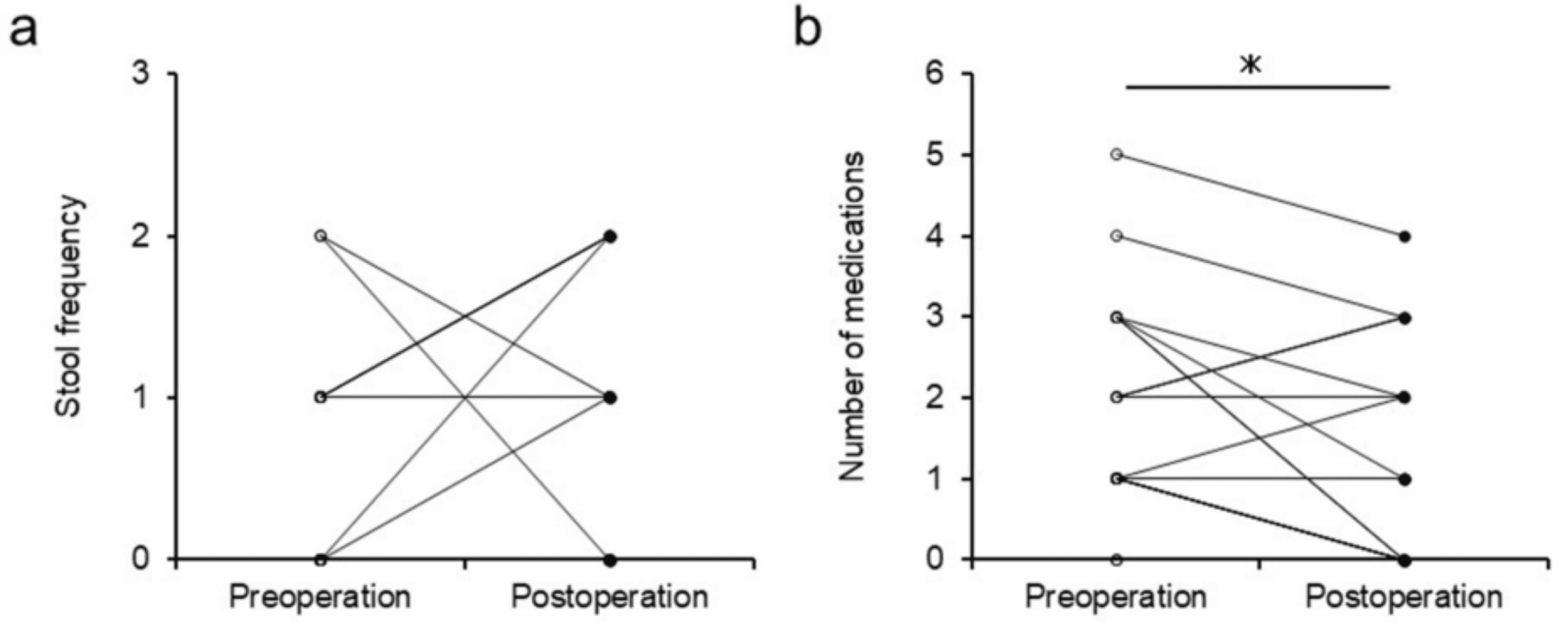
Graphs showing parameters relating to fecal evacuation before and after surgery (a) Stool frequency. (B) Number of medications taken for constipation. ^*^ P < 0.05

The alteration of defecation after surgery is summarized in Figure 5.

Stool frequency was not significantly different before and after surgery, whereas the number of medications for constipation was significantly lower after surgery compared with before (P < 0.05).

## Discussion

Any findings of ischemic necrosis or perforation in patients with sigmoid volvulus are indications for emergency surgery [6,7,9], and approximately 25% of cases present as emergencies with clinical signs of peritonitis or shock related to ischemia or perforation [7,14]. While endoscopic decompression and detorsion are commonly performed for nonemergency cases, with subsequent elective prophylactic surgery recommended, most patients with sigmoid volvulus are elderly, neuropsychologically impaired, or institutionalized and have multiple comorbidities [2]. These frail patients often have poor surgical tolerance, and clinicians are frequently reluctant to perform elective prophylactic surgery. The rate of complications following elective surgery has been reported to be 57.4%, with a mortality rate of 0-3.3% [8,15,16]. Elective surgery is recommended for patients classified as American Society of Anesthesiologists physical status I-III, as it is associated with lower mortality and longer survival, even in those with severe systemic disease [16].

Similar to previous reports, the majority of participants in our study were poor surgical candidates, with past medical histories of cerebral stroke, Parkinson’s disease, or fractures causing reduced activity, and many participants were institutionalized [8,17]. In this study, elective prophylactic surgery caused no serious complications or deaths and was performed safely even in frail patients.

Prophylactic surgery after endoscopic treatment is recommended during the same hospital admission [7], typically one to three days after endoscopic treatment [18]. In our study, some cases did not undergo prophylactic surgery during the first hospitalization because clinicians preferred to delay surgery, favoring conservative management until recurrence occurred. While this approach may reflect real-world clinical practice, since sigmoid volvulus is a nonmalignant disease and patients are often poor surgical candidates, patients with recurrent volvulus face considerable mortality risk [8]. Recurrence among patients who have not undergone surgical resection carries the greatest mortality risk, exceeding 20% [17,18]. Our study showed that one of 10 patients (10%) with recurrent volvulus died from the condition.

The nutritional status of patients with recurrent volvulus worsened with subsequent hospitalizations, which, to our knowledge, is the first study comparing nutritional condition across multiple hospitalizations. Undernutrition, including low preoperative PNI, has been reported to be associated with postoperative complications among patients with colon cancer [12]. Our results support the recommendation that prophylactic surgery should be performed at the first onset of sigmoid volvulus.

The decision to restore colonic continuity is at the discretion of the attending surgeon and is typically based on the patient’s clinical stability [3]. Sigmoid colectomy is common in prophylactic surgery, whereas Hartmann’s procedure is considered safest for emergency surgery [3]. Restoration of continuity is optimal in the absence of peritoneal contamination, and studies have shown no difference in mortality between Hartmann’s procedure and resection with anastomosis [15]. Regarding nutritional condition, patients with PNI >45 can safely undergo resection and anastomosis of the gastrointestinal tract, whereas PNI 40-45 carries considerable risk, and PNI <40 may be a contraindication [19]. In our study, patients with preoperative PNI 40-45 and <40 accounted for 29% and 42% of the population, respectively, but all underwent sigmoid colectomy safely without anastomotic leakage.

An elongated colon in volvulus is often associated with failure of agglutination of the mesentery with the parietal peritoneum [20]. Consequently, the redundant colon is not fixed to the dorsal wall and can swing freely. During surgery for sigmoid volvulus, the sigmoid colon can usually be exteriorized through a small incision (Figure 1) and resected. Thus, laparoscopic sigmoid colectomy is suitable in most cases, as observed in participants who underwent elective surgery in this study.

Patients with sigmoid volvulus typically have a longer sigmoid colon and wider mesosigmoid, while the width of the mesosigmoid root is comparable to individuals without volvulus [21]. The incidence of redundant colon ranges from 1.9% to 28.5%, and colon transit time increases with redundant colon length, causing constipation, bloating, and abdominal pain [20]. In cases with redundant colon and slow-transit constipation, colectomy improves defecation and yields high patient satisfaction [22]. In our study, the number of medications for constipation decreased after surgery, which likely reflects improved management of fecal evacuation. Stool frequency did not change significantly, suggesting that reduced medication use is a marker of improved bowel function. This benefit complements the prevention of recurrence.

This study has several limitations. First, it was a single-center retrospective study with a small sample size. While we reviewed medical records carefully, it is possible that some patients declined surgery because clinicians emphasized surgical risks due to frailty rather than providing detailed evidence of the high recurrence rate and low mortality of prophylactic surgery. Second, obtaining subjective information was challenging, as some patients could not accurately describe their symptoms due to age or sequelae of stroke; thus, we evaluated objective indicators, such as stool frequency and medication use for constipation. Third, recurrence may have occurred in patients lost to follow-up, potentially underestimating recurrence rates. Fourth, deterioration of nutritional status before recurrence was likely due to multiple factors, including aging, chronic disease, and inflammation, but the exact mechanisms remain unclear.

## Conclusions

Sigmoid volvulus frequently recurs in patients managed without surgical treatment, posing a life-threatening risk with each recurrence. Most of these patients are frail due to advanced age and have multiple comorbidities, with nutritional status often deteriorating before recurrence. Therefore, elective prophylactic surgery should be planned promptly after the first endoscopic treatment. Delaying prophylactic surgery can lead to unfavorable preoperative conditions, including further deterioration of nutritional status at the time of recurrence. In addition to preventing recurrence, surgery for sigmoid volvulus can improve the management of fecal evacuation.
